# Characterization and genomic analysis of two *Aeromonas* phages

**DOI:** 10.3389/fmicb.2025.1585026

**Published:** 2025-06-09

**Authors:** Li ling Jiang, Chang Liu, Guang feng Liu, Xiu hua Tao, Hong Sai Zhang, Yue Zhang, Yuyu Chen, Meng yao Li, Jing zhe Jiang, Hong peng Chen

**Affiliations:** ^1^School of Traditional Chinese Medicine, Guangdong Pharmaceutical University, Guangzhou, Guangdong, China; ^2^Key Laboratory of South China Sea Fishery Resources Exploitation and Utilization, Ministry of Agriculture and Rural Affairs, South China Sea Fisheries Research Institute, Chinese Academy of Fishery Sciences, Guangzhou, Guangdong, China; ^3^Institute of Forest Medicinal Materials and Food, Jiangxi Academy of Forestry, Nanchang, Jiangxi, China

**Keywords:** *Aeromonas* phages, biological characteristics, genomic analysis, phylogenetic tree, antibacterial curve

## Abstract

Phages, as viruses that specifically infect certain bacteria, have great research value in the prevention and control of bacterial diseases. Bacteria of the genus *Aeromonas* are widely distributed in aquatic environments and are important zoonotic pathogens that can cause a variety of diseases. In this study, two virulent phages, AhC3_1 and AsC4_1, were successfully isolated using bacteria from the genus *Aeromonas*. The evolutionary relationships of the two phages were analyzed using methods such as phylogenetic analysis and average amino acid identity (AAI) analysis, and their biological characteristics were assessed through approaches including one-step growth curve assay and temperature tolerance testing. The results showed that the genome sequences of phages C3 and C4 were 232,884 bp and 45,983 bp in length, respectively, with G + C contents of 44.36% and 50.11%. Both belong to double-stranded circular DNA viruses. Phages C3 and C4 have been classified within two novel viral genera, provisionally designated as “Aynavirus” and “Asnavirus,” respectively. Additionally, both C3 and C4 exhibited good stability in different temperature and pH conditions. The latent period of C3 was 10 min, with a burst size of 32 PFU.cell^−1^. The in vitro antibacterial curve showed that when the multiplicity of infection (MOI) was 10^−3^, C3 could exert a strong inhibitory effect on *Aeromonas* hydrophila for up to 10 h. The latent period of C4 was less than 10 min, with a burst size of 1,250 PFU.cell^−1^. At an MOI of 10^−1^, C4 could exert a strong inhibitory effect on *Aeromonas* salmonicida for up to 15 h. In summary, the two phages isolated in this study have the potential to serve as potential therapeutic agents for aquaculture diseases and provide valuable data for research on antibacterial infections.

## 1 Introduction

*Aeromonas* bacteria are Gram-negative bacilli belonging to the family *Aeromonadaceae* (Fernandez-Bravo and Figueras, 2020) and are widely distributed in aquatic environments. They are important zoonotic pathogens (Pan et al., 2022). In humans, infections caused by these bacteria can lead to wound infections, septicemia, pneumonia (Schwartz et al., 2024), and gastroenteritis (Abd El-Ghany, 2023). In aquatic animals, they primarily cause hemorrhagic septicemia, fin rot, diarrhea, enteritis (Woo et al., 2022), and fish furunculosis (Ling, 2020). *Aeromonas hydrophila* and *Aeromonas salmonicida* are the most common pathogenic species within the genus *Aeromonas* and are also common opportunistic pathogens in aquaculture, causing significant losses to the industry.

The misuse and long-term use of antibiotics are significant factors contributing to bacterial resistance and the emergence of multidrug-resistant strains. Antibiotic pollution enables *Aeromonas* bacteria to acquire resistance genes from the environment (Gonzalez-Avila et al., 2021). Studies have shown that *Aeromonas* strains isolated from different sources exhibit varying degrees of resistance to antibiotics such as aminoglycosides, quinolones, trimethoprim-sulfamethoxazole, cephalosporins (Tao et al., 2024), amoxicillin, and tetracycline (Teodoro et al., 2022). Additionally, bacteria have evolved resistance mechanisms under such conditions, such as the production of β-lactamases (Mohanty et al., 2024). Therefore, there is an urgent need to seek new therapeutic approaches. Phage therapy, with its high specificity, environmental friendliness, and ability to kill antibiotic-resistant bacteria, has regained attention.

Phages are a class of viruses that specifically infect and lyse bacteria (Wang R. et al., 2024). They are composed of nucleic acid as the genome and a protein capsid (with rarely an inner membrane) and can generally be divided into virulent phages and temperate phages. Phages are ubiquitous and are the most diverse and abundant biological entities on Earth, holding immense potential for application and development (Sharma et al., 2017). In medicine, the combination of phages and antibiotics can significantly improve antibiotic resistance in bacteria (Petrovic Fabijan et al., 2020). Moreover, phages have various innovative applications in vaccine development, cancer therapy, and as gene delivery vectors (Cui et al., 2024). In agriculture and food safety, phages can be used for disease prevention and health improvement in animals and plants (Żbikowska et al., 2020), as well as for antimicrobial control in food products such as milk and luncheon meat (Pang et al., 2022). Additionally, phages have applications in environmental protection and biotechnology to varying degrees (Monk et al., 2010; Rees and Dodd, 2006). Overall, as naturally occurring viruses with unique characteristics, phages have broad application potential in the fields of medicine, agriculture, food, environment, and biotechnology.

Previous studies have identified bacteriophages that can specifically infect bacteria of the genus *Aeromonas* (Hou et al., 2023; Liu et al., 2020; Pallavali et al., 2023), demonstrating their potential as environmentally friendly agents for controlling *Aeromonas* infections and specifically killing multidrug-resistant strains of *Aeromonas* in medical, food, agricultural, and other public settings. In this study, we report two additional novel bacteriophages, AhC3_1 and AsC4_1, that infect *Aeromonas* spp., and we conducted comparative analyses of their biological and genomic characteristics.

## 2 Materials and methods

### 2.1 Materials

#### 2.1.1 Strains

The host strains used for phage isolation in this study were *Aeromonas hydrophila* and *Aeromonas salmonicida*, which were isolated from bullfrog tissues at a bullfrog breeding farm in Guangzhou, Guangdong Province, China.

#### 2.1.2 Reagents and instruments

Brain Heart Infusion (BHI) (HuanKai Microbiology, Guangdong, China); Agar powder (BioSharp, Anhui, China); SM buffer (YuanYe Bio-Technology, Shanghai, China).

Laminar flow cabinet, incubator shaker, and incubator (YiHeng Scientific, Shanghai, China); Sigma 3-18KS high-speed centrifuge (Sigma, Lower Saxony, Germany).

### 2.2 Preparation of host bacterial suspension

The host bacteria stored at –80°C were inoculated into BHI liquid medium and cultured overnight at 28°C. The bacterial suspension was then transferred to fresh BHI liquid medium at a ratio of 1% and cultured until the logarithmic phase. The culture was stored at 4°C for later use.

### 2.3 Isolation and verification of phages

Water samples were collected from a commercial bullfrog (*Lithobates catesbeianus*) aquaculture farm located in Guangzhou, Guangdong Province, China. The samples were concentrated through membrane filtration to enhance bacteriophage recovery. Subsequently, two novel bacteriophages were successfully isolated using the double-layer agar method (Gao et al., 2023), with two previously characterized bacterial strains (*Aeromonas hydrophila* C3 and *Aeromonas salmonicida* C4) serving as host cultures. The cross-titration assay employing the double-layer agar method (Gao et al., 2023) was conducted to verify potential reciprocal infectivity between the two phages, wherein 1 mL aliquots of exponential-phase host bacteria (*Aeromonas hydrophila* C3 and *Aeromonas salmonicida* C4) were separately mixed with 6 mL semi-solid BHI medium (0.7% agar) in 15 mL centrifuge tubes, followed by pouring the mixtures onto petri dishes, air-drying for approximately 10 min, performing quadrant streaking, and subsequently spot-titrating 10 μL purified phage onto designated sectors of each plate. The bacteriophage suspension in SM buffer was purified through sequential double-layer agar plating (3–4 cycles) until homogeneous, translucent plaques were obtained. A single plaque was then excised and inoculated into exponential-phase host bacterial culture for overnight amplification. Phage viability and titer were subsequently verified by the standard double-layer agar method. For long-term preservation, the filtered (0.22 μm) purified phage was mixed 1:1 (v/v) with a modified SM buffer-based cryoprotectant (containing 15% glycerol), aliquoted, and stored at –80°C. Parallel samples were maintained at 4°C for short-term usage.

### 2.4 Genome sequencing and bioinformatics analysis

A total of 30 mL of the host bacterial culture was grown to the logarithmic phase, and 200 μL of phage suspension (≥ 10^7^ PFU/mL) was added. The mixture was then cultured overnight. The bacterial and phage mixture was directly sent to Magigene Biotechnology Co., Ltd in Guangdong for sample pre-processing and sequencing. The sequencing was performed using next-generation sequencing (NGS) technology on the Illumina NovaSeq 6000 (Illumina, San Diego, California) platform. The genome sequences were assembled using Megahit (v1.1.2) (Li et al., 2016). Viral sequences were identified through the following bioinformatics pipeline: First, assembled contigs (assemble. final. contigs) were compared against the CheckV database (v1.0) (Nayfach et al., 2021), with contigs shorter than 600 bp or of low confidence being removed. Subsequently, clean reads were aligned to the target viral contigs using BWA (v0.7.17) and SAMtools (v1.8), requiring a minimum alignment length of ≥ 80% of the read length. The average sequencing depth for each contig was then calculated. These combined analyses enabled comprehensive phage sequence identification. All viral genomes obtained in this study have been deposited in GenBank with the accession numbers: PV158446 [AsC4_1] and PV158447 [AhC3_1].

The complete phage genome sequences were submitted to PhageScope^1^ (Wang R. H. et al., 2024) to construct the genomic map. Information on the predicted conserved proteins (terminase large subunit, capsid protein major head protein) in the phage genome was obtained.

Conserved protein sequences were compared using BLASTP (Altschul et al., 1990; Altschul et al., 1997) against the Non-redundant protein sequences (nr) database, with an Expect threshold (e-value) set at 10^–5^, and the top ten sequences with the highest Max Score were downloaded. All viral nucleotide sequences of the order *Caudoviricetes* (as listed in the International Committee on Taxonomy of Viruses, ICTV, MSL39. V3) were downloaded. The ORFs of these *Caudoviricetes* phage sequences were predicted using Prodigal (Hyatt et al., 2010) (V2.6.3), resulting in a set of ORFs (referred to as the ICTV_pro set). The conserved proteins of the phages were then compared against the ICTV_pro set using Diamond (version 0.9.14.115) BLASTP, and the matching sequences were downloaded. These sequences were combined with the results obtained from the NCBI BLASTP and outgroup sequences. After removing redundancy, the sequences were aligned using the Muscle algorithm. A neighbor-joining phylogenetic tree was generated using MEGA 7 (version 7.0.26) with 1,000 bootstrap replicates (Saitou and Nei, 1987). The phylogenetic tree was visualized using iTOL (Letunic and Bork, 2021)^2^. All sequences selected for phylogenetic tree construction based on the conserved TerL gene were subjected to multiple sequence alignment using EMBL-EBI Job Dispatcher (Madeira et al., 2024). The average amino acid identity (AAI) was calculated from these alignments. Visualization of AAI results was performed by generating heatmaps through the chiplot online platform^3^.

Based on phylogenetic analysis of the conserved TerL gene in phages C3 and C4, we compared their genomes with 4–5 representative viral genomes from their two closest evolutionary relatives (selected from the two most closely related genera) using VIPtree (v1.9) (Nishimura et al., 2017)^4^.

The complete genome sequences were uploaded to PhageScope^1^ (Wang R. H. et al., 2024), and homology searches were conducted using mmseqs in the VFDB and CARD databases to identify virulence factors and antibiotic resistance genes. For predicting the lifestyle of the phages, the Graphage tool was used to distinguish between virulent and temperate phages.

### 2.5 The optimal multiplicity of infection (MOI)

Fresh logarithmic-phase host bacterial liquid culture was prepared, and the concentration of the bacterial suspension was fixed. The concentration of the phage was adjusted to achieve Multiplicity of Infection (MOI, phage/host bacterium) values of 10, 1, 0.1, 0.01, 0.001, 0.0001, and 0.00001. For each MOI, 100 μL of the mixture was added, a blank control was established by preparing a mixture containing 100 μL of host bacterial suspension and 100 μL of BHI broth culture medium. mixed thoroughly, and then allowed to stand for 10 min. Subsequently, 1 mL of BHI broth was added, and the mixture was incubated in a shaking incubator at 28°C for 6 h. The titer of the phage was then determined using the double-layer agar method. Three replicates were performed for each experimental group.

### 2.6 One-step growth curve assay

A total of 1 ml of the purified phage was mixed with the logarithmic-phase host bacterial suspension at the optimal multiplicity of infection (MOI). A blank control was established by preparing a mixture containing 100 μL of host bacterial suspension and 100 μL of BHI broth culture medium. The mixture was incubated at 28°C for 15 min. After incubation, the mixture was centrifuged at 10,000 rpm/min for 1 min to remove the unadsorbed phages in the supernatant. The supernatant was discarded, and 1 ml of BHI broth was added to the pellet. The mixture was then incubated in a shaking incubator at 28°C.

Samples were collected every 10 min for the first 30 min, every 15 min from 30 to 150 min, and every 30 min thereafter. The phage titers were determined using the double-layer agar method. Three replicates were performed for each experimental group.

The burst size was calculated using the formula:

Burst size = (Phage titer at the end of lysis-Phage titer at the beginning of lysis)/Host bacterial concentration at the start of infection.

### 2.7 Temperature and pH stability

For the temperature treatment groups, 500 μL of purified phage was added to 2 mL centrifuge tubes and incubated at different temperatures (4°C, 28°C, 37°C, 50°C, 65°C, and 80°C) for 30, 60, and 90 min. The phage titers under different temperature treatments were determined using the double-layer agar method. Three replicates were performed for each experimental group.

The pH stability of purified bacteriophage was assessed by preparing SM buffer solutions across a pH range of 1–13 using acid (HCl) and base (NaOH) reagents. For each test condition, 900 μL of pH-adjusted SM buffer was mixed with 100 μL of purified phage and incubated in a shaking incubator at 28°C for 1 h. The phage titers under different pH treatments were measured using the double-layer agar method. Three replicates were performed for each experimental group.

### 2.8 *In vivo* antibacterial curve determination

Logarithmic-phase host bacterial suspension was mixed with purified phage at different Multiplicity of Infection (MOI) ratios (with the host bacterial concentration fixed and the phage concentration adjusted). In a 96-well plate, 100 μL of host bacterial suspension and 100 μL of purified phage were added to each well to achieve the desired MOI ratios. A blank control with 100 μL of BHI broth and a negative control with 100 μL of host bacterial suspension plus 100 μL of BHI broth were also set up. Each group was run in triplicate. The plate was incubated at 28°C for 45 h in a microplate reader, with OD600 measurements taken every hour.

### 2.9 Statistical analysis

The software used for data processing, analysis, and graphing includes Excel, Origin 2024, and SPSS. The error bars represent the standard deviation of the experimental data.

## 3 Results

### 3.1 Morphological observation of phages

**FIGURE 1:**
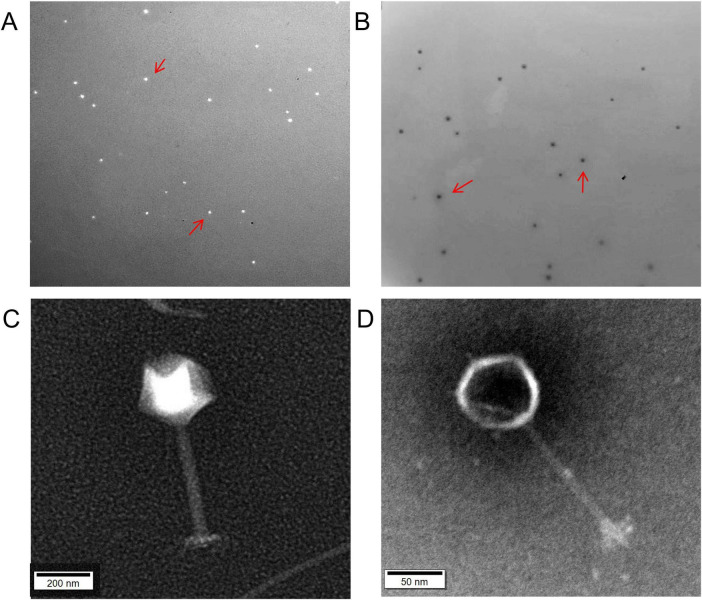
Phage plaque morphology and electron microscopic characteristics of phages C3 and C4. **(A)** Plaque of C3, with a central diameter of approximately 0.5 mm, appearing as a transparent small dot with clear edges. **(B)** Plaque of C4, with a central diameter of approximately 1 mm, appearing as a transparent dot with fuzzy edges. **(C)** Transmission electron microscopy (TEM) morphology of phage C3. The virion exhibits an icosahedral head approximately 142 nm in diameter, attached to a slender tail measuring about 223 nm in length. **(D)** TEM morphology of phage C4. This phage displays an icosahedral capsid with a diameter of approximately 54 nm, connected to an elongated tail structure measuring roughly 121 nm.

Using *Aeromonas hydrophila* and *Aeromonas salmonicida* as host bacteria, two phages, AhC3_1(C3) and AsC4_1(C4), were isolated from aquaculture water samples. Cross-titration assays demonstrated strict host specificity, with lytic plaques exclusively forming at the application sites of phage C3 on *Aeromonas hydrophila* C3 plates and phage C4 on *Aeromonas salmonicida* C4 plates, confirming the absence of reciprocal infectivity and establishing these as two distinct novel bacteriophages. The plaques formed by the phages were observed using the double-layer agar method (Figure 1). The plaques of C3 appeared as transparent, small dots with clear edges and a diameter of approximately 0.5 mm (Figure 1A). In contrast, the plaques of C4 were transparent dots with fuzzy edges and a diameter of about 1 mm (Figure 1B). Transmission electron microscopy (TEM) analysis revealed that (Figures 1C, D), Phage C3 exhibiting an icosahedral head approximately 142 nm in diameter and a long, non-contractile tail with a total length of about 223 nm. Phage C4 featuring an icosahedral capsid measuring approximately 54 nm in diameter and an elongated, non-contractile tail approximately 121 nm in length.

### 3.2 Bioinformatics analysis of phages

#### 3.2.1 Genomic map

**FIGURE 2:**
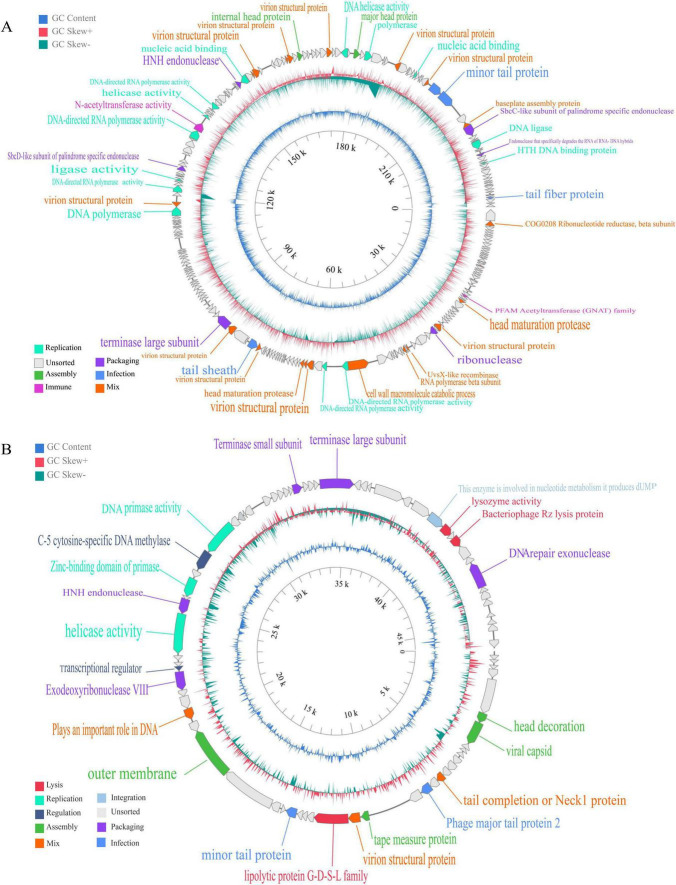
Genomic structure of the two phages C3 and C4. **(A)** C3; **(B)** C4. From the innermost to the outermost region: (1) The numbers represent the genome length; (2) The blue circle indicates deviations in GC content; (3) The circle composed of red and green represents GC skew; (4) The outermost arrows represent open reading frame (ORF), which are distinguished by their direction and color, and are annotated based on their functions. Proteins of different functional modules are indicated by different colors in the legend at the lower left corner of the image. The phage genome map was constructed using PhageScope (Wang R. H. et al., 2024).

After whole-genome sequencing and bioinformatics analysis, the genome sequences of phages C3 and C4 were found to be 232,884 bp and 45,983 bp in length, respectively, with G + C contents of 44.36% and 50.11%. Both are classified as double-stranded circular DNA viruses. The genome annotation of C3 revealed 237 open reading frames (ORFs), 189 hypothetical proteins, and 48 ORFs with known functions. Among these, six ORFs are related to packaging functions, two to phage assembly, 14 to DNA replication, four to phage infection of the host, two to phage immunity, and 20 ORFs are associated with proteins of mixed functions (Figure 2A). All viral genomes obtained in this study have been deposited in GenBank with the accession numbers: PV158446 [AsC4_1] and PV158447 [AhC3_1].

The genome annotation of C4 revealed 77 ORFs, 54 hypothetical proteins, and 23 ORFs with known functions. Among these, five ORFs are related to packaging functions, four to phage assembly, three to DNA replication, three to phage lysis of bacterial cell walls, one to phage-encoded functions, two to phage regulation, two to phage infection of the host, and three ORFs are associated with combinations of two different functions (Figure 2B).

No resistance genes or virulence genes were detected in the phage genomes.

#### 3.2.2 Phylogenetic, whole-genome level, average amino acid identity analysis

**FIGURE 3:**
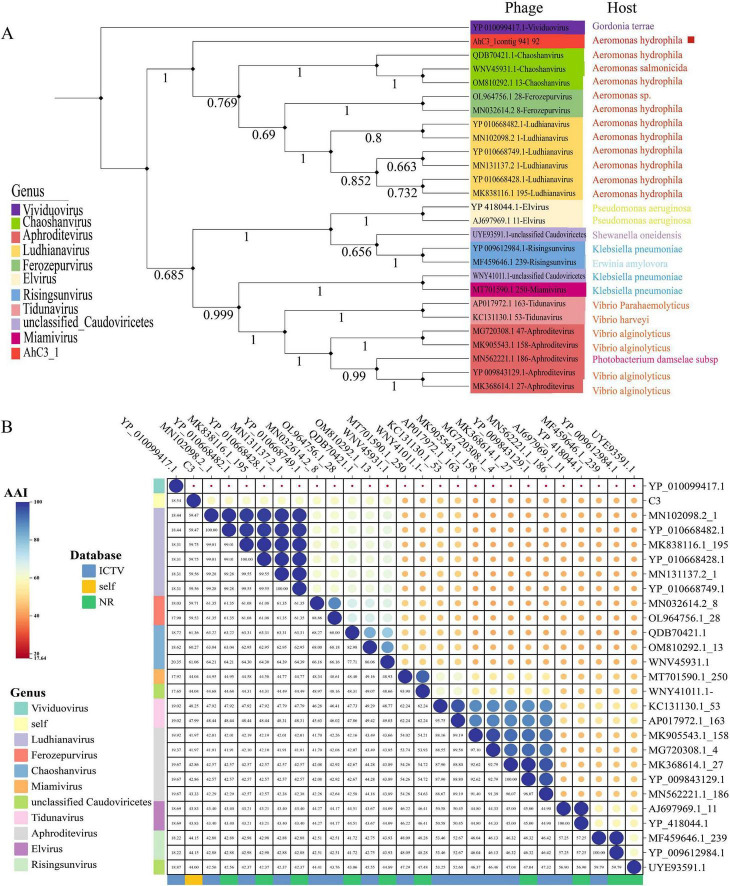
Phylogenetic and average amino acid identity analysis of phage C3 based on terminase large subunit. **(A)** Neighbor-joining phylogenetic tree of phage C3 constructed using the terminase large subunit (TerL). The target phage is marked with a red square. Different genera are represented by distinct colors as indicated in the legend. Sequences belonging to the genus Vividuovirus served as the outgroup, with sequence origins differentiated by color in the legend. The tree was generated using MEGA 7 (version 7.0.26) with 1,000 bootstrap replicates (Saitou and Nei, 1987). Multiple sequence alignment was performed using the MUSCLE algorithm. **(B)** Average amino acid identity (AAI) analysis of phage C3 based on the conserved TerL gene. The target phage is highlighted with a red square. The color gradient represents AAI values, with dark blue indicating the highest identity and red the lowest. Different genera are color-coded as shown in the legend. AAI analysis was conducted using the EMBL-EBI Job Dispatcher (Madeira et al., 2024).

**FIGURE 4:**
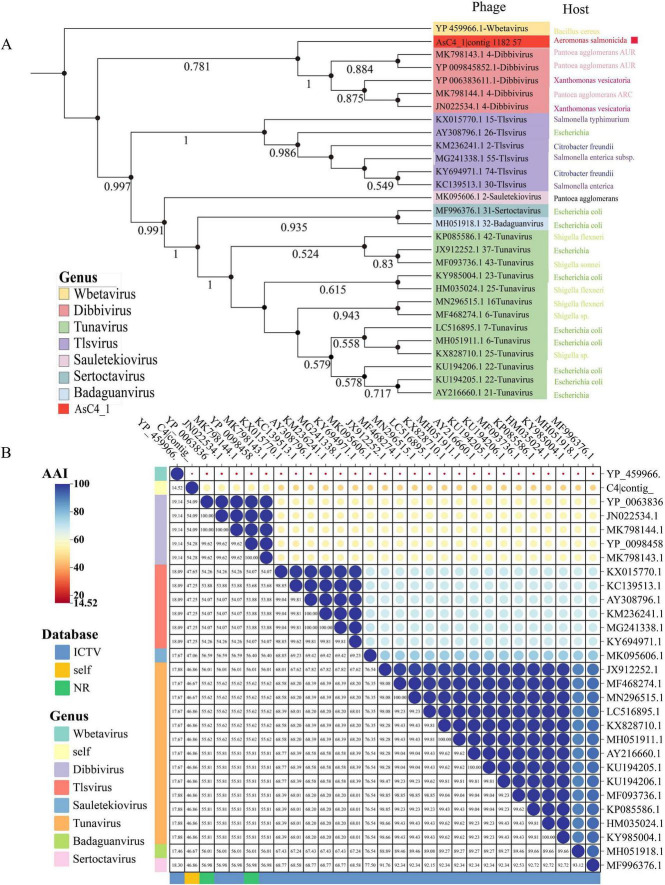
Phylogenetic and average amino acid identity analysis of phage C4 based on terminase large subunit. **(A)** Neighbor-joining phylogenetic tree of phage C4 constructed using the terminase large subunit (TerL). The target phage is marked with a red square. Different genera are represented by distinct colors as indicated in the legend. Sequences belonging to the genus Wbetavirus served as the outgroup, with sequence origins differentiated by color in the legend. The tree was generated using MEGA 7 (version 7.0.26) with 1,000 bootstrap replicates (Saitou and Nei, 1987). Multiple sequence alignment was performed using the MUSCLE algorithm. **(B)** Average amino acid identity (AAI) analysis of phage C4 based on the conserved TerL gene. The target phage is highlighted with a red square. The color gradient represents AAI values, with dark blue indicating the highest identity and red the lowest. Different genera are color-coded as shown in the legend. AAI analysis was conducted using the EMBL-EBI Job Dispatcher (Madeira et al., 2024).

Phylogenetic analysis of phages C3 and C4 was performed using MEGA 7 (Saitou and Nei, 1987), with neighbor-joining trees constructed for each. For phage C3, phylogenetic trees based on two conserved genes - the terminase large subunit (TerL) and major capsid protein - both demonstrated closest evolutionary relationships with members of the *Chaoshanvirus* and *Ferozepurvirus* genera (Figure 3A and Supplementary Figure 1A). In the case of phage C4, phylogenetic reconstruction using TerL revealed closest evolutionary proximity to the *Dibbivirus* and *Tlsvirus* genera (Figure 4A). However, analysis based on the capsid protein gene showed different phylogenetic affiliations, with closest relationships to unclassified *Caudoviricetes* phages and the *Feofaniavirus* genus (Supplementary Figure 1B).

Based on phylogenetic analysis of the conserved terminase large subunit (TerL) genes from phages C3 and C4, we aligned their genomes with 4–5 representative viral genomes from their closest evolutionary relatives (selected from the two most closely related genera for each phage) using VIPtree (Nishimura et al., 2017 and Supplementary Figures 2A, B). The analysis revealed significant conservation of TerL sequences in both phages compared to their closest relatives, with sequence identity ranging between 40% and 70%.

The average amino acid identity (AAI) analysis of all conserved TerL sequences used for phylogenetic tree construction was performed for phages C3 and C4 using EMBL-EBI Job Dispatcher (Madeira et al., 2024). As shown in Figure 3B, phage C3 exhibited the highest AAI values (60%–61%) with members of the genus *Chaoshanvirus*. Similarly, Figure 4B demonstrates that phage C4 showed maximal AAI (approximately 54%) with viruses from the genus *Dibbivirus*. According to established taxonomic criteria proposed by Lavigne et al. (2009), an AAI threshold > 70% is required for classification within the same genus. Given that both *Chaoshanvirus* and *Dibbivirus* currently lack higher taxonomic classification at the family level, and considering the complementary phylogenetic evidence from TerL analysis, we propose the establishment of two novel genera: Phage C3 as the type species of genus “*Aynavirus*”; Phage C4 as the type species of genus “*Asnavirus*.”

### 3.3 Optimal multiplicity of infection (MOI) and one-step growth curve

**FIGURE 5:**
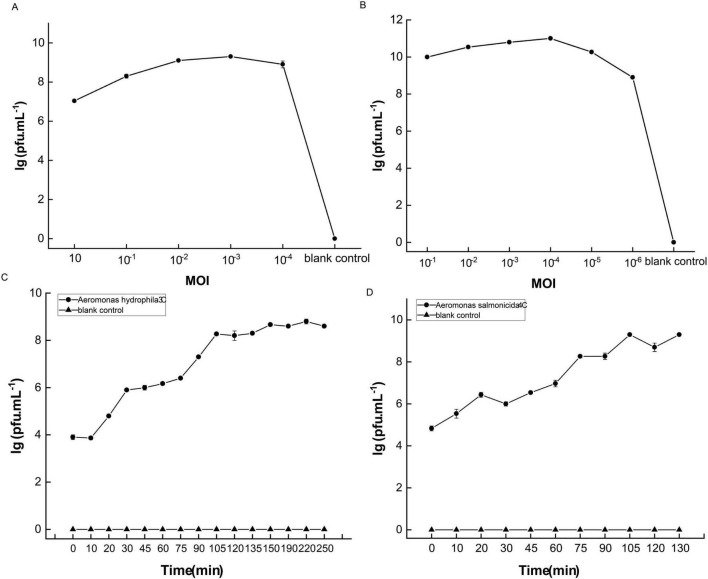
Optimal multiplicity of infection (MOI) and one-step growth curves of the two phages C3 and C4. **(A,B)** Optimal MOI of C3 and C4, respectively. **(C,D)** One-step growth curves of C3 and C4, respectively. Pfu stands for plaque-forming unit. Blank control consisting of host bacteria without phage.

As shown in Figure 5A, the optimal Multiplicity of Infection (MOI) for phage C3 is 10^–3^, while the optimal MOI for phage C4 is 10^–4^ (Figure 5B). One-step growth curves were established using these optimal MOI ratios. From the one-step growth curves of the phages (Figure 5C), it can be seen that the latent period of C3 is 10 min, after which the titer increases exponentially and stabilizes after 150 min, with a burst size of 32 PFU/cell. For C4, the latent period is less than 10 min, with the titer beginning to rise exponentially within 10 min and stabilizing after 105 min, maintaining stability thereafter, with a burst size of 1,250 PFU/cell (Figure 5D).

### 3.4 Temperature and pH stability

**FIGURE 6:**
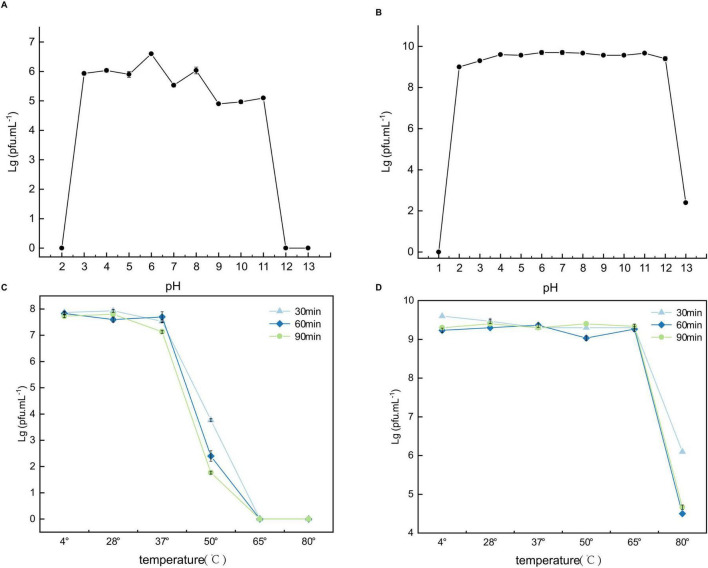
Stability of the two phages C3 and C4 at different temperatures and pH levels. **(A,B)** pH stability of C3 and C4, respectively. **(C,D)** Temperature stability of C3 and C4, respectively. Pfu stands for plaque-forming unit.

The stability of phages under different pH conditions was observed using the agar plate method after 60 min of pH treatment. As shown in Figure 6A, phage C3 remained stable between pH 3 and 11, losing activity in highly acidic (pH 2) or highly alkaline conditions (pH 12 and 13). Figure 6B indicates that phage C4 exhibited good stability between pH 2 and 12, maintaining a consistent titer range. Activity decreased in highly alkaline conditions (pH 13) and was lost in highly acidic conditions (pH 1). These results demonstrate that both C3 and C4 have strong tolerance to acidic and alkaline conditions.

The temperature stability of phages was also observed using the agar plate method under different temperature treatments. Figures 6C, D show that the impact of temperature on both phages was minimal across different treatment durations (30, 60, and 90 min). Phage C3 maintained stability between 4°C and 37°C, with a decline in activity above 37°C and loss of activity above 65°C (Figure 6C). Phage C4 remained stable between 4°C and 65°C, with a decrease in activity above 65°C (Figure 6D). This indicates that C4 has a broader temperature tolerance range compared to C3.

### 3.5 *In vitro* antibacterial curve result

**FIGURE 7:**
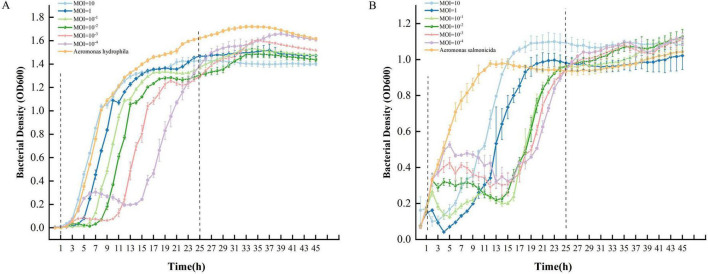
*In vitro* bacteriostatic curves of the two phages. **(A)** *In vitro* bacteriostatic curves of C3 at different multiplicity of infection (MOI). **(B)** *In vitro* bacteriostatic curves of C4 at different MOI. The yellow line represents the bacteria-only negative control.

The antibacterial effects were evaluated over a 45 h period under controlled *in vitro* conditions using various multiplicity of infection (MOI) ratios of *Aeromonas hydrophila*-phage C3 mixtures, and different MOI ratios of *Aeromonas salmonicida*-phage C4 mixtures. As shown in the *in vitro* antibacterial curves (Figure 7A), within the interval enclosed by the two black dashed lines, phage C3 exhibited a strong inhibitory effect on the host bacteria for 10 h at an MOI of 10^–3^, indicating that C3 had a better antibacterial effect at this MOI compared to other ratios within this interval.

Similarly, within the interval enclosed by the two black dashed lines, phage C4 showed a strong inhibitory effect on the host bacteria for 15 h at an MOI of 10^–1^ (Figure 7B), indicating that C4 had a better antibacterial effect at this MOI compared to other ratios within this interval.

## 4 Discussion

The genus *Aeromonas* belongs to the family *Aeromonadaceae*. As important pathogens, these bacteria pose varying degrees of health risks to humans and can also cause a variety of diseases in aquaculture. In this study, two unidentified *Aeromonas* phages were isolated from aquaculture water and characterized and analyzed genomically. From the genomic features and evolution of the phages, both *Aeromonas* phages belong to the order *Caudoviricetes*. In this study, phylogenetic analysis based on TerL and the major capsid protein consistently indicated that C3 exhibits the closest evolutionary distance to the genera *Chaoshanvirus* and *Ferozepurvirus* (Figure 3A and Supplementary Figure 1A). For C4, the TerL-based phylogenetic tree suggested its closest evolutionary affinity with the genera *Dibbivirus* and *Tlsvirus* (Figure 4A), whereas the capsid protein-based tree placed it closer to unclassified *Caudoviricetes* phages and the genus *Feofaniavirus* (Supplementary Figure 1B). The observed discrepancies in the phylogenetic relationships inferred from different conserved proteins of the same phage strain may be attributed to variability in the evolutionary rates among phage proteins. TerL plays a key role in recognizing the initiation and termination sites of DNA packaging, cutting DNA, and participating in the DNA packaging process in phages. This high specificity and critical function make TerL highly conserved among different phages (Hilbert et al., 2017). The most widely and highly conserved signature protein in *Caudoviricetes* is the terminase large subunit (Cai et al., 2019; Lokareddy et al., 2022; Sørensen et al., 2020). Therefore, this study selected the large terminase subunit (TerL) as the phylogenetic marker to construct evolutionary trees for the two *Aeromonas* phages. Based on the amino acid identity (AAI) analysis of TerL, phages C3 and C4 were proposed as novel genera, designated “*Aynavirus*” and “*Asnavirus*,” respectively. With the discovery of additional *Aeromonas* phages in the future, the taxonomic framework for this phage group is expected to be further refined.

The virulence genes carried by phages can not only directly enhance the pathogenicity of bacteria but also increase the risk of dissemination of these genes through gene transfer and environmental spread, which can significantly reduce the safety of their applications. Therefore, comprehensive and clear genetic and evolutionary information of phages can be obtained through whole-genome sequencing and genomic analysis of phages, and measures such as selecting virulent phages can provide safety guarantees for phage therapy. Importantly, no resistance genes or virulence genes were detected in the phage genomes in this study, further supporting the suitability of the two *Aeromonas* phages as candidates for phage therapy.

The temperature and pH stability of phages are key factors for their important roles in the food industry, medical field, and environmental applications. By selecting phages with broad temperature and pH tolerance ranges, their reliability and effectiveness in applications can be enhanced. Some reported *Aeromonas hydrophila* phages, such as *Aeromonas hydrophila* phages AP-T65, AP-T5, AP-Y28, and AP-AT (Ture et al., 2022), remain active between 4°C and 37°C, with a significant decrease in activity above 50°C. The pH range for high activity of AP-T65, AP-T5, AP-Y28, and AP-AT is 3–10. Another example is the *Aeromonas salmonicida* phage ASG01 (Li et al., 2024), which remains active between 30°C and 60°C, with its activity dropping to 0% at 70°C. The optimal pH range for ASG01 is 4–12. In this study, the *Aeromonas hydrophila* phage C3 remains active between 4°C and 37°C, with a high activity pH range of 3–11. The *Aeromonas salmonicida* phage C4 maintains infectivity up to 65°C, with a significant decrease but still detectable activity above 80°C. The pH range for high activity of C4 is 2–12. Compared with the aforementioned reported phages, phages C3 and C4 exhibit relatively higher thermal and pH stability. Therefore, understanding and controlling these characteristics are of great significance for the application of phages.

The *Aeromonas salmonicida* phage C4 can produce lytic proteins, which is a significant advantage for its application. In this study, the presence of phage lytic proteins was identified in the genome of the *Aeromonas salmonicida* phage C4. Lytic proteins can increase the number of phages released from bacterial cells, thereby enhancing the phage’s burst capacity. Similar findings have been reported in other studies, such as the vB_AsM_ZHF phage, where the presence of phage lytic proteins was also detected, and its burst capacity was found to be strong (Xu et al., 2021). Phage lytic proteins are encoded by phages and specifically degrade the bacterial cell wall, allowing the phage to be released from the infected bacteria. As a potential antimicrobial agent, phage lytic proteins have a broader spectrum of activity than the phage itself, making them highly valuable for research (Bai et al., 2020; Lu et al., 2023). Another type of phage lytic enzyme, produced as a soluble free enzyme, is called endolysin. Studies have shown that endolysin LysZC1, obtained from the Pseudomonas phage ZCPS1, exhibits the highest bactericidal activity against Bacillus cereus (Abdelrahman et al., 2023). This highlights the significant potential of phage C4 in antibacterial applications.

The strong antibacterial capability is another significant advantage of phage applications. Analysis of the *in vitro* antibacterial curves of phages can intuitively show the inhibitory effect of phages on host bacteria, as well as the growth of resistant bacteria. In this study, we plotted antibacterial curves at different Multiplicity of Infection (MOI) levels and found that both *Aeromonas phages* can cause high-intensity, sustained inhibition (lasting 10 h) of the host bacteria at specific MOI values. Similar findings have been reported for three *Aeromonas* phages (ZHA, ZHD, and ZHF) (Xu et al., 2021), which also exhibited acceptable, sustained antibacterial effects (lasting 25 h) on host bacteria at specific MOI values. The *Aeromonas* phage MQM1 (Hosseini et al., 2023) also showed good antibacterial activity against its host bacteria. These results suggest that the strong antibacterial effect of *Aeromonas* phages on their host bacteria may be a common phenomenon and indicate that the two *Aeromonas* phages have potential applications in treating bacterial infections.

Phages with potent antibacterial activity represent a crucial prerequisite for bacteriophage-based applications. As naturally occurring viruses with unique biological properties, phages exhibit broad application potential across diverse fields, including medicine, agriculture, food safety, environmental remediation, and biotechnology. Compared to conventional antibiotics, phages offer distinct advantages such as high host specificity, self-replication capability, and environmental friendliness, making them promising alternatives for combating antibiotic-resistant infections. However, as phage applications expand, studies have revealed that bacteria can develop resistance through various evolutionary mechanisms [e.g., altering phage receptor sites or increasing mutation rates (Sharma et al., 2017)], thereby compromising therapeutic efficacy and limiting their widespread use. To address these challenges, current research primarily focuses on the following strategic approaches: Isolation of broad-host-range phages: Screening phages capable of infecting multiple bacterial strains to minimize bacterial escape; Phage cocktail therapy: Employing multiple phages in combination to expand host coverage and reduce resistance development, rather than relying on a single phage; Rescue of phage activity via mutagenesis: Selecting or artificially inducing mutant phages from wild-type populations to restore infectivity against resistant bacteria (Labrie et al., 2010); Development of engineered phages: Designing or modifying phages to enhance their targeting efficiency against resistant microbes (Kilcher and Loessner, 2019); Combined antimicrobial strategies: Integrating phages with antibiotics, antimicrobial peptides, or other antibacterial agents to synergize efficacy and delay resistance emergence. These strategies not only improve the reliability and effectiveness of phage applications but also provide critical directions for optimizing and commercializing phage-based therapies in the future.

In summary, the two *Aeromonas* phages isolated in this study exhibit distant phylogenetic relationships with all currently known phages, representing the first members of the proposed new genera “*Aynavirus*” and “*Asnavirus*.” This finding provides important taxonomic references for newly discovered phages. Both phages demonstrate specific lytic activity against *Aeromonas* strains, coupled with remarkable thermal and pH stability, indicating strong environmental adaptability that supports their potential applications in complex settings (e.g., aquaculture and food processing). However, this study has several limitations that warrant further investigation: The current host spectrum remains unexplored; future studies should evaluate lytic efficacy against diverse *Aeromonas* species and related pathogens to delineate host specificity and application scope. While *in vitro* antibacterial activity has been confirmed, establishing animal infection models (e.g., zebrafish or turbot) is essential to systematically assess therapeutic efficacy and potential toxicity for clinical translation. Although morphological (TEM) and genomic features have been characterized, the structural proteome remains unannotated. Subsequent work should employ SDS-PAGE coupled with LC-MS/MS to identify key components (e.g., major capsid and tail fiber proteins), elucidating host recognition mechanisms and enabling targeted genetic engineering. These follow-up studies will bridge the gap between fundamental discovery and practical applications of these novel phages.

## 5 Conclusion

In this study, using *Aeromonas hydrophila* and *Aeromonas salmonicida* as host bacteria, two phages, C3 and C4, were isolated from aquaculture water samples. The analysis revealed that these phages have high titers and are stable across a broad range of temperatures and pH levels. No virulence or antibiotic resistance genes were detected in their genomes. Phylogenetic analysis revealed that both phage C3 and phage C4 represent putative novel genera within the class *Caudoviricetes*. Additionally, both *Aeromonas* phages exhibited strong antibacterial capabilities, with C4 encoding lytic proteins. These characteristics indicate the potential of these two phages as therapeutic agents for treating aquaculture diseases.

## Data Availability

The original contributions presented in this study are publicly available. This data can be found here: https://www.ncbi.nlm.nih.gov/, accession numbers: PV158446 and PV158447.

## Footnotes

1
https://deepomics.org

2
https://itol.embl.de

3
https://www.chiplot.online/

4
https://www.genome.jp/viptree/

## Supplementary material

Supplementary Figure 1 Phylogenetic analysis of phages C3 and C4.

Supplementary Figure 2 Whole-genome comparative analysis of phages C3 and C4.
