# Supplementary figures and images for: Characterization and genomic analysis of two Aeromonas phages
Source: Front Microbiol. 2025 Jun 9;16:1585026. doi: 10.3389/fmicb.2025.1585026 (PMC12184794; doi:10.3389/fmicb.2025.1585026)

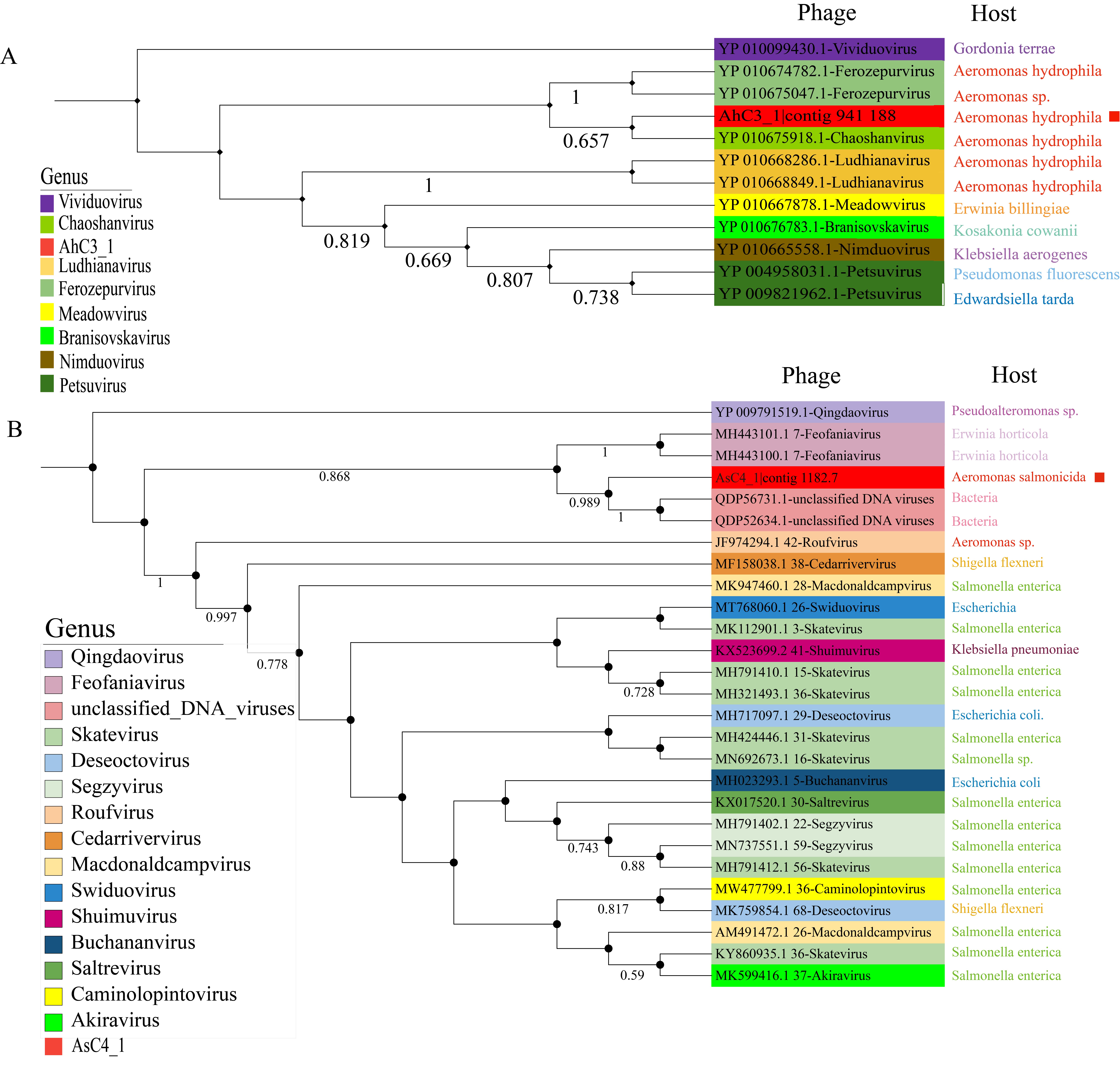

Supplement: Supplementary Figure 1 — Phylogenetic analysis of phages C3 and C4. [file Image_1.jpeg]
